# Disease Status of Afghan Refugees and Migrants in Pakistan

**DOI:** 10.3389/fpubh.2019.00185

**Published:** 2019-07-03

**Authors:** Muhammad Suleman Malik, Muhammad Afzal, Alveena Farid, Fati Ullah Khan, Bushra Mirza, Mohammad Tahir Waheed

**Affiliations:** ^1^Department of Biochemistry, Faculty of Biological Sciences, Quaid-i-Azam University, Islamabad, Pakistan; ^2^Department of Animal Science, Faculty of Biological Sciences, Quaid-i-Azam University, Islamabad, Pakistan; ^3^Ayub Teaching Hospital, Abbottabad, Pakistan

**Keywords:** Afghan refugees, internally displaced people, Pakistan, disease status, healthcare, disease-related mortality

## Abstract

World is facing the largest refugee crisis of its time due to continuously outgoing wars, conflicts and natural disasters. One of the important aspects of refugees and migrants is health. Till date, no comprehensive data was available related to health status of Afghan refugees and internally displaced persons (IDPs) in Pakistan. Here, we present health status for Afghan refugees for last seven years and for IDPs for 2–4 years. For Afghan refugees the data was provided by Commissionerate Afghan Refugee (CAR), Pakistan, whereas data for IDPs was collected from hospitals and Basic health units (BHUs) of different districts of Khyber Pakhtunkhwa namely Peshawar, Dera Ismail Khan and Bannu. Highest number of Afghan refugee's deaths occurred due to cardiovascular problems. Most prevalent reported infections were respiratory tract infections (48.05%). Skin diseases and Diarrhea collectively affected 21.08% of Afghan refugees. Overall, disease burden was more in females than males in Afghan refugee's population. To the best of our knowledge, this is the first comprehensive report on health and disease status of Afghan refugees and IDPs in Pakistan.

## Introduction

Pakistan is a developing country, holding sixth largest population in the world with a number reaching ~208 million according to a recent census conducted by Pakistan Bureau of Statistics (1). Pakistan is ranked among low-middle income countries (LMIC) by World Bank (2), having ~4$ per day income (3). It is estimated that 39% of Pakistani population is living in multidimensional poverty (4), marked by several factors, mainly poor health, lack of education, less income, poor quality of work and threat of violence. Approximately, 65% of Pakistani population is residing in rural areas where health, hygiene and sanitation conditions are not very good compared to urban areas. These factors make the residing population at risk of various infections and diseases. Most migrants or refugees prefer to live in rural areas due to less costs of living. Also, in most cases, the camps arranged by the government for living of refugees lie in outskirts of urban areas. This increases the burden of already populated rural areas and thus incidence of diseases increases. Additionally, movement of people from one place to other serves as a source of dispersal of infections to new areas, where previously those infections were absent or less prevalent.

Recently, United Nations High Commissioner for Refugees (UNHCR) reported that there were 22.5 million refugees at the end of the year 2016 around the world (5). This number may have been increased in 2017 due to number of new crisis that resulted in massive migration such as recent movement of Rohingya people from Myanmar to Bangladesh. As per this report (5), Pakistan was ranked as second-largest refugee hosting country (1.6 million) after Turkey (2.5 million). Around 2.3 million Afghan refugees came to Pakistan within years 1979–1982 as they fled from war with Soviet Union (6). In addition to influx of refugees, Pakistan has been facing the crises of internally displaced people (IDPs) from time to time because of military operations against terrorism and conflicts (7). Throughout the history, one of the major problems of humanity has been the migration especially for developing countries (8). Regardless of efforts that were made by World Health Organization (WHO), UNHCR, non-governmental organizations (NGOs) and other humanitarian organizations, basic needs of refugees such as housing, food and other social needs are often not met. One of the major problems that refugees face is accessibility to medical facilities and this situation becomes more critical when it comes to developing countries (9).

When refugees transit from non-endemic region to an endemic region, they are more prone to local diseases as compared to indigenous population, as they are not immune to native strains (10). The communicable and non-communicable disease burden is double on Pakistan as it is passing through epidemiological transition (11). A communicable disease is an infection that is transmissible to persons from animate or inanimate sources whereas non-communicable diseases (NCD: chronic diseases) have the tendency to be of long duration and are the aftereffect of a mix of different factors (genetic, physiological, environmental and behavioral) (12, 13). In Pakistan, ~38% of deaths occur due to communicable, maternal, perinatal and nutritional conditions and non-communicable diseases accounts for 51% of total deaths whereas remaining 11% of deaths occur due to injuries (14). NCD includes diabetes, chronic respiratory diseases (CRD), cardiovascular diseases and cancer and risk factors associated with them (15).

Pakistan has been facing terrorism, economic problems, political instability, poverty and health problems from several decades (16–18). Among these problems, health has been a major issue. Pakistan is prone to disasters such as earthquakes, floods and drought (19). Due to continuously ongoing war in Afghanistan since last four decades, there has been constant influx of Afghan people to Pakistan for shelter, refuge and sometimes for business. Additionally, ~1.8 million Pakistani people were internally/temporarily displaced due to military operations against terrorism in 2015 (20). All these factors collectively affect the health status of afghan refugees and temporarily displaced persons and put indigenous population at risk. There has been lack of data regarding overall health status of afghan refugees and IDPs in Pakistan in recent past. This data is necessary for the Government and related-organizations to carryout health-care activities and target certain diseases. The main objective of this report was to collect the data and evaluate the disease status of the afghan refugees and IDPs in Pakistan based on the data collected from different areas/hospitals, government institutes within Pakistan and from online data available from January 2012 to December 2018. The data was processed through a database in MS Excel and represented in the form of graphs and narrations.

## Methods

### Data Collection

**Figure 1:**
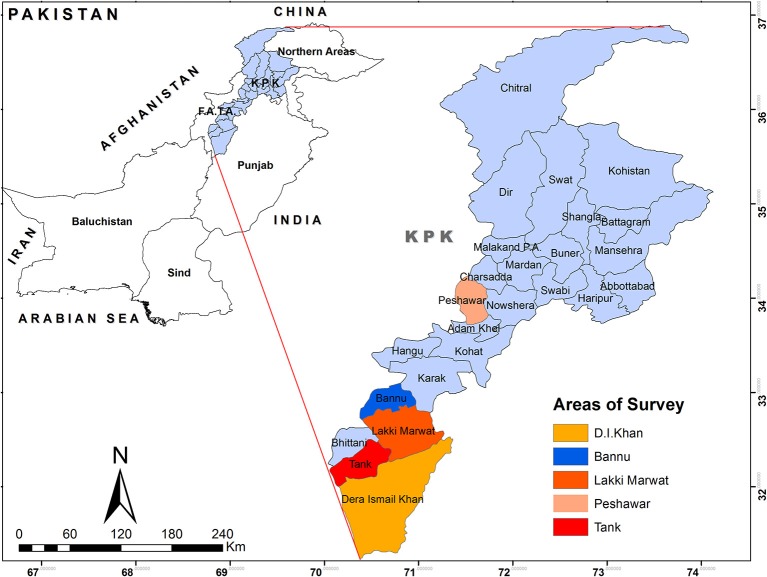
Map of Districts visited for collection of Data. The colored areas show the districts where most Afghan refugees and IDPs were located. KPK, Khyber Pakhtunkhwa province, province adjacent to Afghanistan.

Current study was aimed to evaluate the health status of the Afghan refugees and IDPs in Pakistan. Data for Afghan refugees was collected from Commissionerate Afghan Refugees (CAR). Khyber Pakhtunkhwa (KPK), Pakistan. Data provided by CAR covered 16 Basic Health Units (BHUs), One Sub Health Unit and Implementing Partners (IPs) from January 2012 to Dec 2018. Data comprised of demography, mortality and morbidity against different infectious and non-infectious diseases. In addition, maternity, vaccination and malarial status was also included in Afghan refugee's data. For collection of health record of internally displaced people (IDPs), we surveyed different districts of Khyber Pakhtunkhwa namely Peshawar, Dera Ismail Khan, Bannu, Lakki Marwat, and Tank as shown in Figure 1. Being neighboring areas of Federally Administered Tribal Areas (FATA), these districts contained most population of IDPs. We collected data of communicable and non-communicable diseases of IDPs' enrolled patients from different healthcare centers of IDPs' lodging districts. Data was collected from province KPK because it holds the largest population of Afghan refugees (62%) and IDPs (21). However, there were some limitations, we were unable to collect reliable data of IDPs' health status from Tank and Lakki Marwat due to lack of reliable record in district health centers.

## Results

### Afghan Refugees Health Status

#### Demography

**Figure 2:**
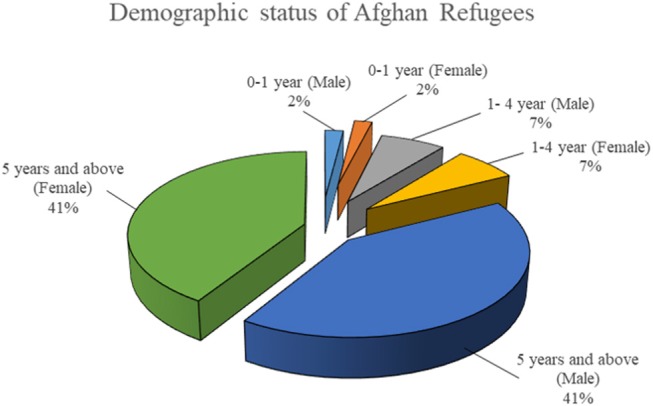
Demographic status of Afghan refugees for year 2012 to 2018.

Figure 2 shows the demographic status of Afghan refugees for last 7 years. The Afghan refugees' population had 41% males and 41% females, with 1:1 ratio of male to female. Among them, 4% of population was under the age of 1 year and 14% between the age of 1–4 years. Rest of population was above the age of 5 years.

#### Mortality and Causes of Mortality

Most patients were males as compared to female and mortality rate was high in males as shown in the Table 1. The major factor for high mortality rate could be due to fact that males are more exposed to external environmental stresses as compared to Afghan females. Per every 1,000 afghan refugees total mortality rate in males was found to be 0.09 and among females it was 0.07. Whereas, below 5 years of age, rate of mortality was 0.24 per 1,000. The main causes of mortality in Afghan refugees (Table 1) were cardiovascular disorders (23.53%) and respiratory disorders (14.22%). Among other causes of mortality were hepatitis (2.75%), watery diarrhea (1.61%), dysentery (1.30%), typhoid (0.74%), measles (0.17%), and tuberculosis (0.12%).

Table 1Mortality and causes of mortality in Afghan refugees.

**Table:** 

**Gender**	**Age**	**2012**	**2013**	**2014**	**2015**	**2016**	2017^*^	2018^*^	**Total deaths**		**Total mortality rate/1000**	
**MORTALITY**												
Male	0–1	90	116	131	88	83	10	17	535	4767	0.01	0.09
	1–4	57	61	87	71	70	88	116	550		0.02	
	5 & above	722	827	799	701	615	18	—	3682		0.06	
Female	0–1	83	96	86	94	64	12	23	458	3762	0.01	0.07
	1–4	73	55	63	84	74	85	88	522		0.02	
	5 & above	546	554	600	556	510	16	—	2782		0.05

**Table:** 

**Years**	**2012**	**2013**	**2014**	**2015**	**2016**	**2017**	**2018**
**CAUSES OF MORTALITY**							
Respiratory disease	219	215	265	228	219	No detail available for causes of mortality	
Watery diarrhea	15	16	44	49	6		
Dysentery	73	2	13	8	9		
Measles	1	1	7	3	2		
Cardiovascular	380	386	403	391	336		
TB	4	2	1	3	0		
Hepatitis	36	45	56	47	38		
Typhoid	12	15	14	14	5		
Others	831	1027	963	851	801		

* *NGO's record is absent*.

#### Morbidity

About 49.12% of Afghan refugees were suffering from respiratory tract infections. Upper respiratory tract infections (URTI) incidence rate (12.74/1000) was significantly high as compared to lower respiratory tract infections (LRTI) incidence rate (3.42/1000). The incidence rates for diarrhea and dysentery were 3.49 and 1.26 out of 1000 afghan refugees, respectively. Skin diseases affected afghan refugees at a rate of 3.59 out of every 1000 refugees. Incidence rate for cardiovascular disorders among afghan refugees recorded was 0.95/1000. Other diseases reported were typhoid (0.25/1000, measles (0.005/1000), and reproductive tract infections (0.94/1000). Out of 9530 Afghan refugees suffering from psychological disorders, 301 patients were referred to psychiatrist. Approximately 42.02% were male patients and 71.42 were female patients. Disease burden and morbidity with respect to age and gender is shown in Table 2.

Table 2Disease morbidity of Afghan refugees.

**Table:** 

**Disease**		**2012**	**2013**	**2014**	**2015**	**2016**	**2017**	**2018**
URTI	Morbidity	144218	129574	154373	179813	137007	6873	1009
	Incidence	16.06	14.82	17.35	21.06	16.75	2.73	0.40
LRTI	Morbidity	46745	30101	32738	50587	39862	1962	64
	Incidence	5.20	3.44	3.68	5.92	4.87	0.78	0.03
Diarrhea	Morbidity	34253	35155	45783	44963	40015	2521	746
	Incidence	3.81	4.02	5.15	5.27	4.89	1.00	0.29
Dysentery	Morbidity	16423	11244	12584	16744	13740	1341	212
	Incidence	1.83	1.29	1.41	1.96	1.68	0.53	0.08
Skin diseases	Morbidity	38309	39681	40316	47692	42908	1889	510
	Incidence	4.27	4.54	4.53	5.58	5.25	0.75	0.20
Typhoid	Morbidity	1126	1575	1739	2931	3124	–	–
	Incidence	0.13	0.18	0.20	0.34	0.38	–	–
Measles	Morbidity	86	74	14	21	12	–	–
	Incidence	0.01	0.01	0.002	0.002	0.001	–	–
Reproductive tract infection	Morbidity	6044	6480	7079	6507	6816	–	7051
	Incidence	0.67	0.74	0.80	0.76	0.83	–	2.78
Cardiovascular	Morbidity	9168	8587	9342	10990	9338	–	3025
	Incidence	1.02	0.98	1.05	1.29	1.14	–	1.19
Psychological disorder	Morbidity	2121	2028	1544	2027	1810	–	–
	Incidence	0.24	0.23	0.17	0.24	0.22	–	–
Referred to psychiatric		24	67	58	78	74	–	–
Others	Morbidity	57522	82358	87845	82797	88924	13765	–
	Incidence	6.40	9.42	9.87	9.70	10.87	5.48	–

**Table:** 

**Gender/Age/Year**			**2012**	**2013**	**2014**	**2015**	**2016**	2017^*^	2018^*^
**MORBIDITY WITH RESPECT TO GENDER AND AGE**									
Gender	Male	Morbidity	166997	153383	170,135	206664	167532	11253	3038
		% Male patients	48.54	45.06	43.89	47.20	44.27	41.12	24.08
	Female	Morbidity	298489	268198	300,977	365931	298750	17098	4035
		% Female patients	86.75	78.79	77.65	83.58	78.94	62.48	31.98
Age (years)	0–1	Morbidity	54857	45412	54077	66423	48945	3461	728
		% 0–1 patients	15.41	13.09	13.75	14.92	12.76	39.69	5.77
	1–4	Morbidity	118968	106185	126208	151940	123898	7769	1432
		% 1–4 patients	33.42	30.61	32.08	34.14	32.30	60.31	11.35
	5 & Above	Morbidity	291661	269984	290827	254232	293439	17121	4913
		% 5 & above patients	81.92	77.84	73.93	57.12	76.50	12.21	38.94
Repeat Visits			11953	6443	5758	7,237	5095	984	0

*URTI, upper respiratory tract infection; LRTI, lower respiratory tract infection*.

* *NGO's record is absent*.

#### Maternal Health Status Among Afghan Refugees

Table 3 shows the health status of mother and children among immigrants. Still birth ratio was 1.29 for 7 years (Jan 2012–Dec 2018). Crude birth rate reported among refugees was 2.51/1000 population. Average Infant Mortality Rate (IMR: 0–1 years) recoded was 8.21 per 1000 live births. Neonatal Mortality Rate (NNMR) and Maternal Mortality Rate (MMR) reported were 2.33/1000 births and 13.70 per 100,000 births, respectively.

Table 3Mother and child health along with vaccination status among Afghan refugees.

**Table:** 

**Year**		**2012**	**2013**	**2014**	**2015**	**2016**	2017^*^	2018^*^	**Total**
A/N first visits		24270	24771	25671	26059	25671	6136	6204	138782
Delivered women >3 A/N visits		20613	22709	23293	24094	23293	5313	5491	124806
Treated for abortions		120	230	253	435	253	6	42	1339
Deliveries by trained staff		13814	15867	17692	7701	17692	4471	5175	82412
Still births		159	139	532	510	532	15	15	1731
Live Births		22699	24885	24673	10714	24673	6601	6390	120635
Maternal deaths	Accidental/Non-obstetrical	2	2	0	0	0	0	–	4
	Natal	0	0	0	1	0	1	–	2
	Postnatal	4	0	1	0	1	1	–	7
Postnatal visits within 3 days		20200	21893	22447	22953	22447	3448	4180	117568
Neonatal deaths		48	47	39	50	38	40	0	247
Complete Antenatal coverage		90.81	91.26	94.41	224.88	94.41	80.49	85.93	108.88
Still Birth ratio		0.70	0.56	2.11	3.07	2.11	0.23	0.23	1.29
Neonatal Mortality Rate (NNMR)		2.11	1.89	1.58	3.27	1.58	5.91	0.00	0.29
Maternal Mortality Rate (MMR)		26.43	8.04	4.05	9.33	4.05	30.30	–	2.33

**Table:** 

**Vaccine**	**Age (yrs.)**	**2012**	**2013**	**2014**	**2015**	**2016**	2017^*^	2018^*^	**Average**
**VACCINATION STATUS**									
BCG	<1+ >1	26216	26879	26107	27621	23730	6640	6760	143953
Measles	<1+ >1	37336	28281	27240	38789	22624	7864	9146	171280
Polio (All 04 doses)	<1+ >1	102751	42831	38963	108465	31923	26425	25235	376593
DPT/pentavalent^**^ (All 03 doses)	<1+ >1	75815	103387	106633	83604	92965	20314	20920	503638
Tetanus vaccine (1 to 5 doses)	—	59323	26831	26866	59985	23450	20955	21108	238518
Fully immunized	<1	27666	79270	80990	28546	70598	7832	7901	302803

* *NGO's record is absent*.

** *Pentavalent vaccination done in year 2017 and 2018*.

#### Vaccination in Afghan Refugees

Detailed status of vaccination in Afghan refugees is summarized in Table 3. The refugees were vaccinated against Tuberculosis (BCG), Measles, Polio, DPT, Tetanus, Diphtheria, Pertussis (whooping cough). From 2012 to 2018, percentage coverage for BCG and measles was 87.31 and 100%, respectively.

#### Proportion of Afghan Refugees With Tuberculosis

Tuberculosis (TB) was responsible for death of ten immigrants from year 2012 to 2018. Positive cases of TB were more in females as compared to males (54.72 and 45.28%, respectively). Thirty-five cases of defaulters were reported among refugees and 29 cases reported for failure in treatment. Immigrants who successfully overcame TB by completing treatment were 207. Table 4 summarizes the TB status among Afghan refugees.

Table 4Tuberculosis and malarial status among Afghan Refugees.

**Table:** 

**Years**	**2012**	**2013**	**2014**	**2015**	**2016**	2017^*^	2018^*^	**Total**
**TUBERCULOSIS STATUS**								
Total new TB cases	156	98	138	149	No case reported			541
New sputum positive	60	35	35	29				159
New sputum negative	96	63	103	117				379
New sputum positive male	22	16	10	24				72
New sputum positive female	38	19	25	5				87
Patients cured [last sputum clear] (a)	53	30	24	13				120
Treatment completed (b)	73	55	51	28				207
Patients died (c)	4	2	1	19				26
Failure of treatment (d)	7	0	21	1				29
Defaulters(e)	4	8	8	15				35
Transferred out(f)	11	12	130	0				153
Total patients evaluated (a+b+c+d+e+f)	152	107	235	76				570

**Table:** 

**MALARIAL STATUS**									
Deaths due to Malaria		2	0	1	0	0	–	–	3
Total positive		2367	1946	2294	2592	1511	396	474	11580
Gender	Male cases	931	796	820	1025	614	No details available		5056
	Female cases	1436	1150	1474	1567	897			6524
Age (yrs.)	0-1	54	38	37	40	19			188
	1-4	380	287	332	533	275			1807
	5 & above	1933	1621	1925	2019	1217			8715
Species	*P. falciparum*	38	8	114	45	11			216
	*P. Vivax*	2242	1840	2180	2538	1498			10298
	Mixed malaria	87	98	0	9	2			196

* *NGO's record is absent*.

#### Proportion of Afghan Refugees With Malaria

Total of three deaths due to malaria were reported from year 2012 to 2018 among Afghan immigrants as shown in Table 4. Over-all, 10,710 positive cases of malaria were testified among refugees. The proportion of malaria infection was high in refugees older than 5 years (81.37%) and in females (60.91%). Most prevalent species in immigrants was *P. vivax* as compared to *P. falciparum* (96.15 and 2.01%, respectively). Total 196 cases of mixed malaria were reported.

### Internally Displaced Persons (IDPs) Health Status

Tables 5–7 summarizes the status of internally displaced people (IDPs) in three different districts namely Peshawar, Dera Ismail Khan and Bannu. Skin diseases were more common than other disorders/diseases in district Peshawar (3,248 cases) whereas respiratory infections were more common in District Dera Ismail Khan and Bannu (14142 and 9399 cases, respectively). Nineteen cases of polio were reported in district Peshawar in year 2015 and 346 IDPs were referred to psychiatrist. Total of 150 surgeries were performed in district Peshawar and 50 cases of jaundice were reported in district Dera Ismail Khan. Among cardiovascular disorders 2,685 cases (2 years) were reported in District Bannu and 20 cases (in 3 years) in district Peshawar. Two hundred and ninety-three female patients were treated with problems of gynecology. Overall, disease data, including other diseases, is given in Tables 5–7 for District Peshawar, Dera Ismail Khan and Bannu, respectively.

**Table 5:** Health status of IDPs in Peshawar.

**Year**	2014^*^	2015^**^	2016^***^	**Total**
Cardiovascular disorders	8	12	0	20
Causality	406	1533	381	2320
Dental	8	98	0	106
ENT	41	183	0	224
EYE	79	776	0	855
Gynecology	41	252	0	293
Medicine	132	466	1	599
Nephrology	15	425	0	440
Orthopedic	131	1121	0	1252
Pediatrics	144	624	0	768
Pediatric surgery	3	12	0	15
Plastic surgery	1	44	0	45
Psychiatry	11	335	0	346
Skin diseases	120	3128	0	3248
Surgical	27	123	0	150
Polio	–	19	0	19
Respiratory tract infections	12	30	0	42

* Record of 3 months.

** *Record of 6 months*.

*** *Record of 7 months*.

**Table 6:** Health status IDPs in Dera Ismail Khan.

**Year**		2014^*^	**2015**	**2016**	2017^**^
Total no patients	Male	9670	4764	1679	921
	Female	7126	8975	3360	1841
	Children	6834	11421	4293	2352
Common illnesses	G. I. diseases	2156	2204	905	496
	Acute respiratory infection	3036	6776	2797	1533
	Skin diseases	3306	1800	737	404
	Vaccine preventable diseases	1286	1120	457	251
	Fever	6247	6636	2735	1498
	Jaundice	20	16	9	5
	Any other	5709	5240	1224	670
Pregnancy care	Ante natal	1013	816	335	184
	Natal	77	16	6	3
	Post natal	752	536	127	70
	Injury	10	10	–	–
	Deaths	3	3	–	–

* *Record Available for 7 months*.

** *Record Available for 6 months*.

**Table 7:** Health status of IDPs in Bannu.

**Year**		**2014**	**2015**
Total no patients	Male	8160	11665
	Female	6759	9747
	Children	5559	7807
Common illnesses	Cardiovascular disorders	1104	1581
	ENT	2111	2999
	Eye infections	3004	4286
	Neural surgeries	2141	3063
	Orthopedic	3341	4768
	Pediatrics surgery	628	884
	Psychiatric referrals	1361	1947
	Respiratory tract infections	3870	5529
	Skin disease	1934	2756
	Urinary tract infections	984	1406
Pregnancy care	Ante natal	4368	1163
	Natal	642	1195
	Post natal	247	1371
	Injury	10	2
	Deaths	15	20

## Discussion

In the present study, we have collected data from those areas of Pakistan where there was a larger influx of afghan refugees and internally displaced people (IDPs). We have estimated the number refugees and IDPs with illnesses in these areas and assessed their disease status. To our knowledge, this is the first comprehensive report on the disease status of Afghan refugees and IDPs in Pakistan covering different disease and disease-related deaths.

Migration is centuries old phenomenon. People move to other places due to multiple factors such as economic reasons, agricultural crisis, political crisis, natural disasters, wars, and complex emergencies. Later three factors disturb the cultural and social life of migrants or refugees especially the poor ones more than any other factors do, which compels the people to migrate to other places. As the population of world increasing the more people are at risk to be affected by such events (22).

Pakistan has been a victim of terrorism, political and economic instability, and natural disasters such as floods, earthquakes and drought. Some of these factors directly affect the health of people in affected areas and cause displacement of people from one place to other. In addition, poverty, lack of education, poor health-care system and facilities makes people more vulnerable to diseases. More than half population of Pakistan lives in rural areas and is at more risk due to number of additional factors such as non-affordability/non-availability of medicines due to less income, poor sanitation and a smaller number of health care professionals.

A constant war in neighboring Afghanistan since last four decades has directly impacted Pakistan in many ways. Massive migration of people from Afghanistan had an economic impact on an already developing country. Time to time ongoing operations in the border areas result in the displacement of people, largely to neighboring rural areas. This increases the health-related risks of indigenous population. Conversely, migrating people are also at risk to local infections. Till date, no collective data was available regarding disease status of Afghan refugees and IDPs residing in Pakistan in neighboring areas of conflict.

It is often problematic to collect data from remote areas and near-border areas due to military operations and road conditions. Healthcare to indigenous and refugees/IDPs in these areas is provided by local hospitals, military health-care professionals, NGOs, Government-based Basic Health Units (BHUs) and World Health Organization (WHO). It is sometimes very difficult to collect data from all these sources and put it in one form to assess the number of patients and prevalent infections and diseases. In the present study, we have collected data from most of those areas where refugee's influx was highest. Also, these areas were linked to tribal areas (near Afghanistan border) where IDPs were temporarily placed. These areas included districts Peshawar, Dera Ismail Khan, Bannu, Lakki Marwat and Tank from the province of Khyber Pakhtunkhwa, adjacent to Afghanistan. Data from some NGOs, who had discontinued their operation in these areas, could not be retrieved.

We observed 1:1 male to female ration in the present study. Previously, UNHCR survey (23) showed that there were 103 males per 100 females, which is approximately equal to 1:1. During migration, it is expected that a significant number of deaths will occurs whether the migration is done forcefully or voluntarily (22). However, we observed a lower mortality rate. A lower mortality ratio has also been observed in some migrant populations, for example, overall mortality among Vietnamese refugees of England and Wales was very low as it was expected: for males the all-cause standardized mortality ratio was 64 whereas for females it was 56 (24). A previous finding shows that the crude deaths for refugees in Pakistan was 10/10,000 refugees between the year 1998 to 2012 (25). In our study the mortality rate was high for males (0.09/1000) as compared to females (0.07/1000) among afghan refugees. A lower mortality rate could be due to constant health support provided by Government, NGOs, and organizations like WHO.

Risk of various health conditions, like cardiovascular diseases and diabetes, is expected to be more among refugees due to starvation (26, 27). Marshal et al. (28) reported that risk for cardiovascular diseases is more in Cambodian refugees, but proposed an extensive study. In a 2012 report, 10% of afghan refugees in Iran were heart patients (29). In our study, the total rate of cardiac patients is 6.67/1000 in afghan refugees in Pakistan. Stress is the key factor whether the migration is done forcefully, planned or voluntary because it involves the breaking of relation with family, friends, culture and social interactions and to settlement in new and different environment.

We observed rate of psychological disturbances in afghan refugees equivalent to 0.22 per 1,000 persons. There are number of socio-economic factors that could lead to psychological disturbances. Again, males are more prone to such factors and hence are expected to suffer more with psychological problems. One of such factor is language that could be related to mental health (22). However, in case of Afghan refugees in Pakistan, the language may not be a major problem for most of afghan refugees because among them 82% were of Pashtun ethnicity, while rest included Tajiks, Uzbeks, and Persians (23).

Among migrants or refugees, the communicable diseases are not the only the health-related issue which affects them most. Especially the reproductive health of female refugees is of importance too that has to be reported (22). Due to pregnancy or child birth related complications, deaths of more than half million women occur each year (30). In this light, aim of UN fifth Millennium Development Goal was kept to improve the maternal health (31). In Afghanistan, the risk of maternal death has been too high in the world. For instance, in city of Kabul, the maternal mortality was 418/100,000 live births during 2002, which significantly decreased by 60% in the year 2011 that is 166/100,000 live births. In 2011, the health facilities were increased by Government. In a recent study, the perinatal death rate among Afghanis was 29 per 1000 live births in Kabul, Afghanistan (32). Providing training to staff related to reproductive health plays important role in decreasing the mortality rate and morbidity related to mother and child (33). A survey done during the year 1999 to 2000 reported that in refugee camps of district Hangu (Khyber Pakhtunkhwa) the health condition for the women was poor. International Rescue Committee (IRC) trained the staff and increased the knowledge in the community regarding maternal health. As a result the maternal mortality rate decreased to 102 per 100,000 live births in 2004 and 67% of deliveries handles by skilled personnel in 2007 (34). Our data shows that 80% of deliveries were handled by trained staff in 2018 whereas average perinatal deaths from year 2012–2013 rate was 17.30/1000 live births. The mortality rate for infants and child as compared to Pakistan among afghan refugees were low in year 2011 (23). The perinatal deaths during our study timeline was lower than that of Afghanis in Kabul during the year 2011, which may be due to better medical facilities in Pakistan than Afghanistan.

Vaccination of children among refugees in Iran has been lower than the Iranian children due to lack of parental attention and lack of awareness toward such programs (35). Among afghan refugees in Pakistan, only one case of Polio was reported in June of 2016 in 2-year-old refusal child refugee who returned from Afghanistan (36). Afghanistan and Pakistan are only two countries where Polio remains endemic (37). The percentage coverage of immunization in children among Afghan refugees was 100% from 2012 to 2018. It was possible due to the efforts of Pakistan Government immunization program.

Pakistan is among top five countries having high rate of TB (38). In 2011, the National Tuberculosis Control Programme (NTP) achieved 64% detection rate for tuberculosis cases in Pakistan (39). In 2000, the rate of tuberculosis in newly arrived refugees (504 per 100,000 person) in United States was significantly high than its national rate (40). The Jordan National TB Program reported 56 cases among Syrian refugees including the 3 multidrug resistant cases (41). We report total of 541 new TB cases in Afghan refugees during years 2012–2015, whereas no case has been reported between 2016 to 2018.

Many parts of Khyber Pakhtunkhwa and Afghanistan are malarial endemic regions (42). Additionally, malarial control remains challenging as it develops resistance against insecticides and antimalarials in use. Migration of 3 million afghan refugees to Pakistan were vulnerable because they settled in malaria endemic region and non-immune to Anopheline breeding (42). In our study, total of 10710 malarial cases were reported from 2012 to 2018 with total of 3 deaths from malaria. *P*. *Vivax* was most prevalent in these reported cases. Surprisingly, only three malaria-related deaths were reported in 7 years period, although number of positive cases was high. This may be due to underreporting of death cause and actual number could be high (42). Overall, the number of diseased persons and deaths could be well high as many cases remain unreported in resource poor settings.

According to UNHCR, Pakistan was ranked second-largest country in terms of hosting refugees. The number reported for Afghan refugees by UNHCR was 1.6 million (5). In the same report UNHCR reported ~1.1 million IDPs, which according to Internal Displacement Monitoring Center was 1.8 million (20). In total, it makes the total population of migrants to 3.4 million, which makes Pakistan by far the largest migrant-hosting country in the world, compared to Turkey with 2.5 million refugee's population (5).

## Conclusions

In the present report, disease status and disease-related deaths of Afghan refugees for past 7 years is presented. For IDPs, 2–4 years data is given. This data would be helpful to develop policies related to healthcare of refugees and migrants. Further, based on the available numbers, Pakistan can be considered as the country hosting the world's largest migrant population and displaced people.

## Data Availability

The raw data supporting the conclusions of this manuscript will be made available by the authors, without undue reservation, to any qualified researcher.

## Author Contributions

MW, BM, and MM conceived the study design, prepared, and finalized the manuscript. MM, FK, AF, and MA collected and analyzed the data. All authors have read and approved the final version of manuscript.

### Conflict of Interest Statement

The authors declare that the research was conducted in the absence of any commercial or financial relationships that could be construed as a potential conflict of interest.
